# Supplementary material for: Perilipin Isoforms and PGC-1α Are Regulated Differentially in Rat Heart during Pregnancy-Induced Physiological Cardiac Hypertrophy
Source: Medicina (Kaunas). 2022 Oct 11;58(10):1433. doi: 10.3390/medicina58101433 (PMC9611277; doi:10.3390/medicina58101433)
Supplement: Supplementary file 1 [file medicina-58-01433-s001.zip › medicina-1891233-supplementary.pdf]

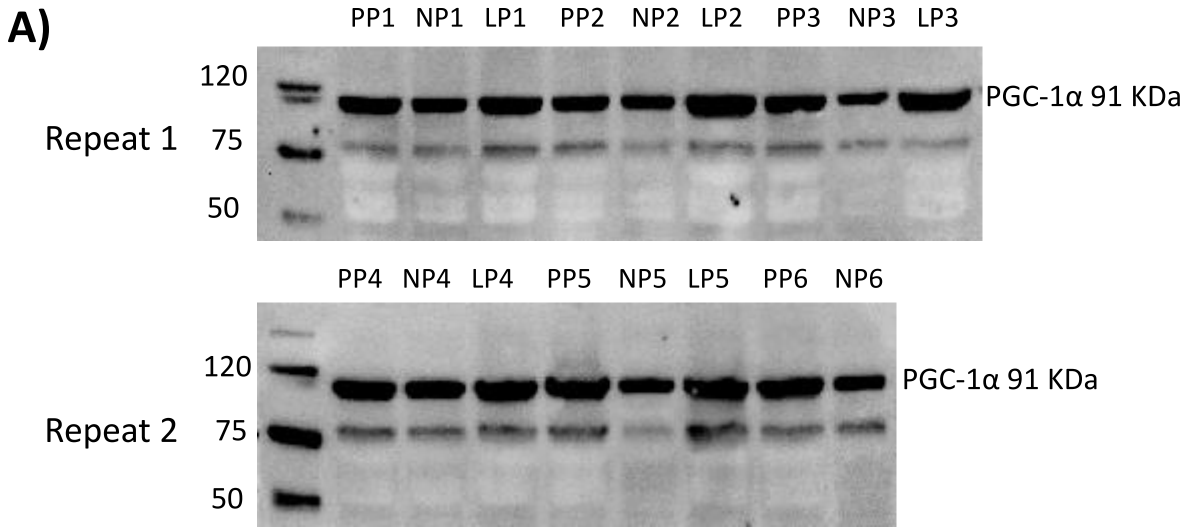

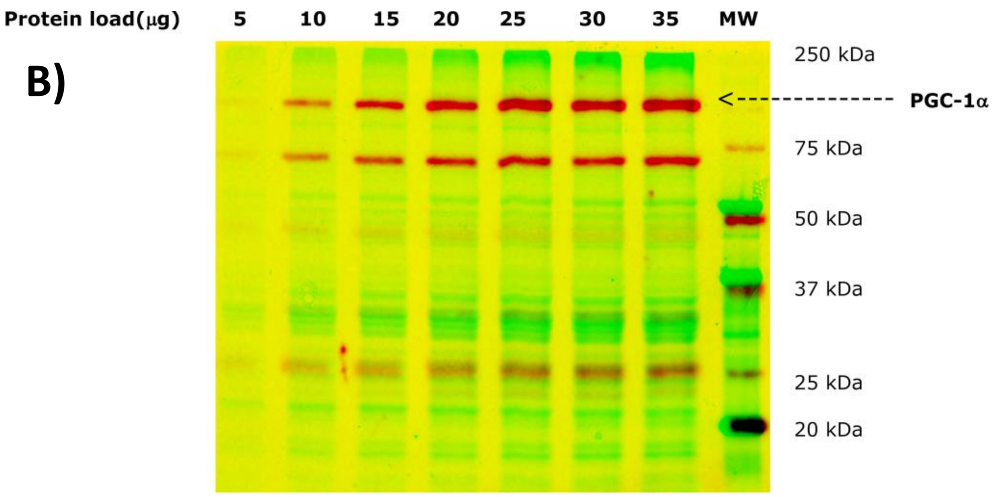

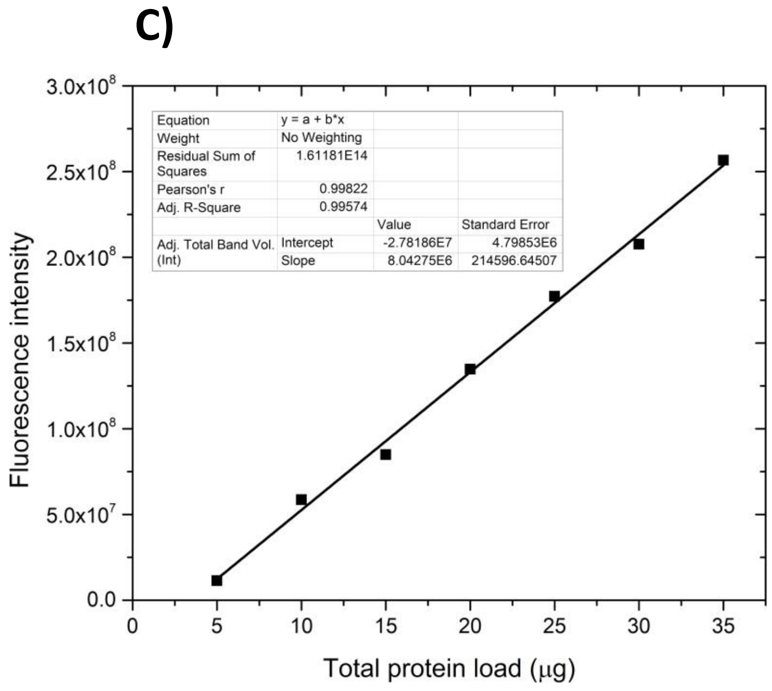

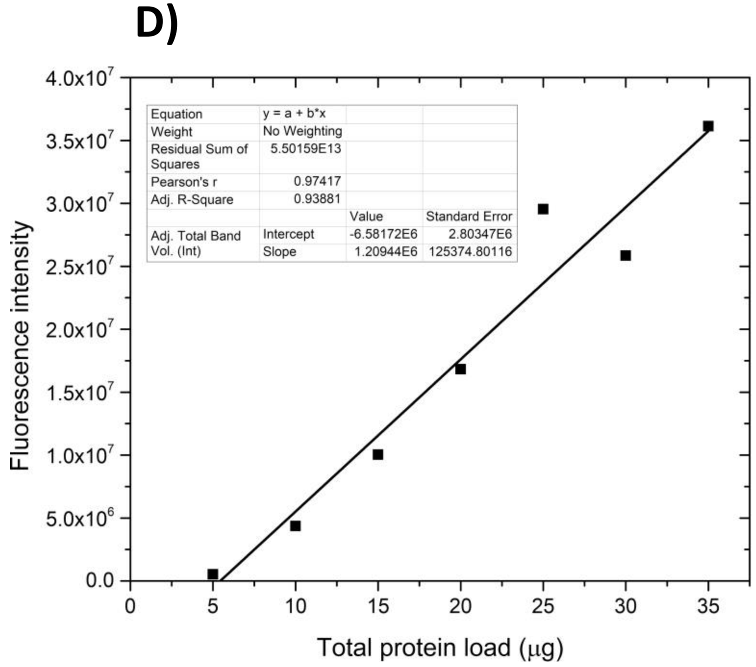

Supplemental Figure S1. PGC-1α western blot normalization analysis. **A.** Blot after cutting membrane at molecular weight 50 KDa, 75 KDa and 120 KDa for PGC-1α (91 KDa) **B.** Electrophoresis analysis in Stain-Free SDS with 5 to 35 μg of total protein load per lane. The stain-free total protein is represented in green and chemiluminescent blot image with fluorescent signal of the PGC-α antibody in red. **C.** Linear fit analysis of total protein load in the working range of 5 to 35 μg. **D**. Correlation analysis of the PGC-1α antibody fluorescent signal with sample concentration.
